# Bioinformatics analysis identifies potential biomarkers involved in the metastasis of locoregionally advanced nasopharyngeal carcinoma

**DOI:** 10.1097/MD.0000000000030126

**Published:** 2022-09-02

**Authors:** Rongrong Hu, Xujun Xu, Lujiao Mo, Mengjie Chen, Yuxiang Liu

**Affiliations:** a Department of Otorhinolaryngology, Zhejiang University Hospital, Hangzhou, China; b Department of Internal Medicine, Zhejiang University Hospital, Hangzhou, China; c Department of Critical Care Medicine, The First People’s Hospital of Xiaoshan District, Hangzhou, China; d General medicine, Community Health Service Center, Dangwan Town, Xiaoshan District, Hangzhou, China; e Department of Critical Care Medicine, The Second People’s Hospital of Xiaoshan District, Hangzhou, China.

**Keywords:** biomarkers, computational bioinformatics, metastasis, molecular biology, nasopharyngeal carcinoma, radiotherapy

## Abstract

Nasopharyngeal carcinoma (NPC) is one of the malignant epithelial tumors with a high metastasis rate. This study aimed to screen potential novel biomarkers involved in NPC metastasis. Microarray data of locoregionally advanced NPC (LA-NPC; GSE103611) were obtained from the database of Gene Expression Omnibus. The differentially expressed genes (DEGs) between LA-NPC tissues with and without distant metastasis after radical treatment were screened. Functional analysis was performed and the protein–protein interaction and submodule were analyzed. The univariate Cox regression analysis was performed to identify prognostic genes in NPC in the validation microarray dataset GSE102349. The drug–gene interactions and key genes were identified. Totally, 107 DEGs were identified. The upregulated DEGs and the key nodes in the protein–protein interaction network were associated with pathways or biological processes related to the cell cycle. Four genes including *CD44*, *B2M*, *PTPN11*, and *TRIM74* were associated with disease-free survival in NPC. The drug–gene interaction analysis revealed that upregulated genes *CXCL10*, *CD44*, *B2M*, *XRCC5*, and *RPL11* might be potential druggable genes for patients with LA-NPC metastasis by regulating cell cycle, autophagy, and drug resistance. Upregulated *CXCL10*, *CD44*, *B2M*, *XRCC5*, and *RPL11* might play important roles in LA-NPC metastasis by regulating cell cycle-related pathways.

## 1. Introduction

Nasopharyngeal carcinoma (NPC) is one of the malignant epithelial tumors arising from the nasopharynx epithelium with a high incidence.^[1,2]^ The global morbidity and mortality of NPC is about 133,000 and 80,000 cases in 2020, respectively.^[1]^ NPC is an Epstein–Barr virus-associated malignancy and has a characterized geographical distribution.^[2,3]^ Over 70% of cases are in the east and southeast parts of Asia.^[2,3]^ Advanced diagnostic tools, sophisticated surgical resection, and conventional pharmacological treatments confer effective prevention and treatment for NPC.^[4]^ Notably, despite a large proportion of patients with locoregionally advanced NPC (LA-NPC) respond well to the above therapeutic methods, patients with secondary or radiation-induced diseases still have an unsatisfactory prognosis and poor survival due to high rates of recurrence and metastasis.^[2,5,6]^ Therefore, it is important to identify novel markers and therapeutic interventions for LA-NPC metastasis.

NPC is characterized by multiple histopathological appearances and complex etiopathogenesis.^[7]^ Cervical lymph node metastasis is the major cause of NPC-related deaths.^[7]^ Numerous characteristic markers of NPC metastasis have been identified by bioinformatics analysis with the revolution of high throughout sequencing technologies, which facilitate early recognition and targeted treatment interventions for NPC metastasis. Using genome-wide methylation microarray data, Ren et al^[8]^ identified aberrantly methylated NPC-specific transcription factors, including the most significantly hypermethylated HOP homeobox HOPX, which promoted NPC metastasis and resulted in poor clinical outcomes. In addition, several research studies showed that genes including matrix metalloproteinase 13,^[9]^ cyclooxygenase-2,^[10]^ flotillin-1,^[11]^ and serine protease inhibitor Kazal-type 6^[12]^ were associated with NPC metastasis. Some of them were associated with the clinical prognosis of NPC.^[8,12–14]^ A recent study based on microarray analysis by Tang and his colleagues^[15]^ established a reliable prognostic 13-gene signature to predict distant metastasis of LA-NPC. The recent biomarkers were limited to improving the diagnosis and clinical therapy of NPC, and further work is still urgently needed to identify potential biomarkers for NPC metastasis, recurrence, and prognosis.

This study aimed to identify potential marker genes associated with LA-NPC metastasis. The microarray data of LA-NPC was used to identify differentially expressed genes (DEGs) between samples from LA-NPC patients with and without distant metastasis. Meanwhile, potential prognostic and druggable DEGs in NPC were identified. This study might provide additional information on the metastasis, development, and treatment intervention of LA-NPC.

## 2. Materials and Methods

### 2.1. Ethical approval

This article does not contain any studies with human participants or animals performed by any of the authors, therefore the ethical approval was not applicable.

### 2.2. Data acquisition

Gene expression microarray dataset GSE103611 was downloaded from the National Center of Biotechnology Information Gene Expression Omnibus database (GEO, http://www.ncbi.nlm.nih.gov/geo/).^[16]^ This dataset is based on the platform of GPL19251 (HuGene-2_0-st) Affymetrix Human Gene 2.0 ST Array (probe set [exon] version). Twenty-four LA-NPC tumor tissues with distant metastasis after radical treatment (metastasis group) and 24 paired LA-NPC tissue specimens without distant metastasis (nonmetastasis group) were included in this dataset.

### 2.3. Data preprocessing and DEGs screening

Data preprocessing was performed using the Oligo package (version 1.36.1, http://bioconductor.org/packages/release/bioc/html/oligo.html) of R software, which mainly consisted of background correction, normalization, and expression calculation. Probe that was not mapped to any gene symbol was removed from our analysis. When, however, 1 gene was mapped by multiple probes, the mean value of the probe was considered as the expression value of this gene. The classical Bayes method provided by the Limma package^[17]^ was used to screen DEGs between the metastasis and nonmetastasis tumors. Significant DEGs were identified according to the criteria of *P* value of <.05 and |log_2_(fold change)| >.5.

### 2.4. Gene Ontology (GO) and KEGG pathway enrichment analyses

To assess functions and significantly enriched pathways of DEGs, GO functional annotation of the biological process^[18]^ and Kyoto Encyclopedia of Genes and Genomes (KEGG) pathway enrichment analysis^[19]^ were conducted with clusterProfiler.^[20]^ The thresholds for significantly enriched biological processes and KEGG pathways were *P* value < .05 and count ≥ 2.

### 2.5. Protein–protein interaction network construction and submodule analysis

The Search Tool for the Retrieval of Interacting Genes/Proteins (STRING) database (version: 10.0, http://www.string-db.org/)^[21]^ that provides the protein–protein interaction (PPI) prediction function is online available. Therefore, we conducted the PPI analysis of DEGs based on this database using the PPI score of 0.15 and expected to identify crucial protein pairs. Afterward, the PPI network was visualized by the Cytoscape software (version 3.2.0, http://www.cytoscape.org/).^[22]^ Moreover, the molecular complex detection (MCODE, version 1.4.2, http://apps.cytoscape.org/apps/MCODE)^[23]^ plugin of Cytoscape was used to analyze submodules with similar functions in the original PPI network. Functional enrichment analysis of GO biological processes and KEGG pathways associated with DEGs were performed using clusterProfiler^[20]^ to investigate the functions of submodules. The top 10 nodes with high degrees in the PPI network and all nodes included in submodules were regarded as hub genes for further analyses.

### 2.6. Screening of validation microarray datasets

To validate the prognostic values and expression patterns of hub genes in NPC tumors, the microarray dataset GSE102349 was downloaded from the GEO database. The microarray dataset was selected according to the following criteria: included of survival data (overall or disease-free survival [DFS] data); included clinical stage; the number of patients was not <20. The GSE102349 dataset (GPL11154, Illumina HiSeq 2000 [Homo sapiens]) included data on DFS, clinical stage, and morphology (differentiated, mixed [round and spindle], and undifferentiated) from 133 patients with NPCs.

### 2.7. Prediction of drug–gene interactions

The Drug–Gene Interaction Database (DGIdb, version 4.2.0; http://www.dgidb.org/)^[24]^ is used to mine existing resources and generate assumptions about how genes are therapeutically targeted or prioritized for drug development. Only FDA-approved drugs were selected to predict drug–gene interactions for hub genes in the PPI network and submodules. Meanwhile, the drug–gene interaction network was constructed and visualized using the Cytoscape software.^[22]^

### 2.8. Literature searching for key genes

GeneCLiP2.0 (http://ci.smu.edu.cn/),^[25]^ a web-based service platform based on literature mining, has 4 major functional modules, including gene function annotation, molecular network construction, GO, and pathway analysis. Literature searching was performed for the druggable genes by Gene Cluster with Literature Profiles. *P* value <.05 and count ≥ 3 were set as thresholds for significant items.

### 2.9. Statistical analysis

The association of hub genes (top 10 nodes in the PPI network and nodes in submodules) with the DFS of NPC was validated in the dataset GSE102349. The univariate Cox regression analysis was performed to investigate the association of hub genes with the DFS of NPC in the GSE102349 dataset. Differences in the expression levels of hub genes across different clinical stages (I/II, III, and IV) and morphologies (differentiated, mixed, and undifferentiated) were analyzed using the nonparametric Kruskal–Wallis H test with Dunn multiple comparisons test. All statistical analyses were carried out using the SPSS software (version 22.0; IBM, Armonk, NY) or the Graphpad Prism software (version 8.3.0; GraphPad software, San Diego, CA). Statistical differences were defined when *P* value was <.05.

## 3. Results

### 3.1. Identification of DEGs

**Figure 1.**
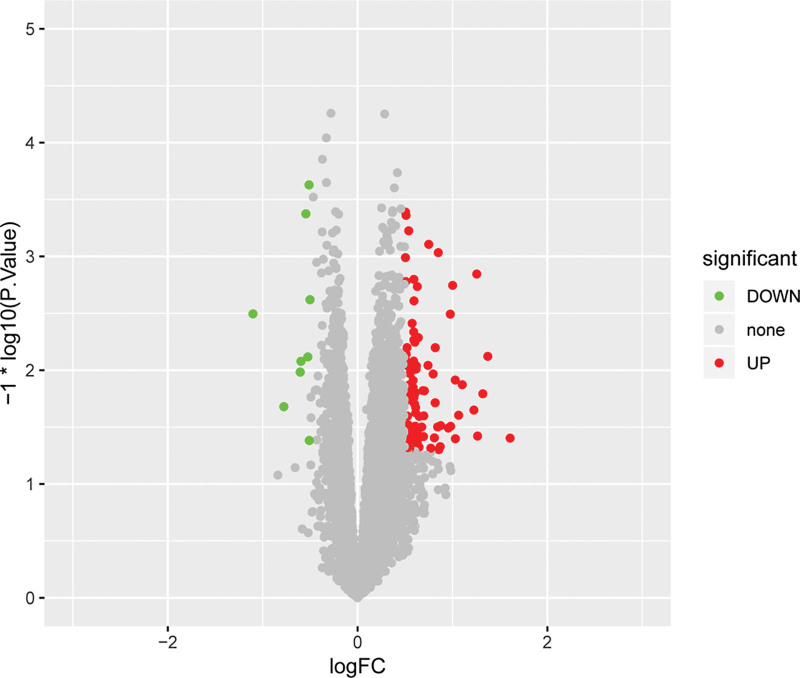
The volcano distribution map of DEGs. Green and red color notes downregulated and upregulated DEGs. DEG = different expressed gene, FC = fold change.

Totally, 107 DEGs between LA-NPC tissue specimens with distant metastasis after radical treatment and LA-NPC tissue specimens without distant metastasis, including 98 upregulated genes and 9 downregulated genes were screened according to the methods described above (Fig. 1).

### 3.2. Functional enrichment analysis of DEGs

**Figure 2.**
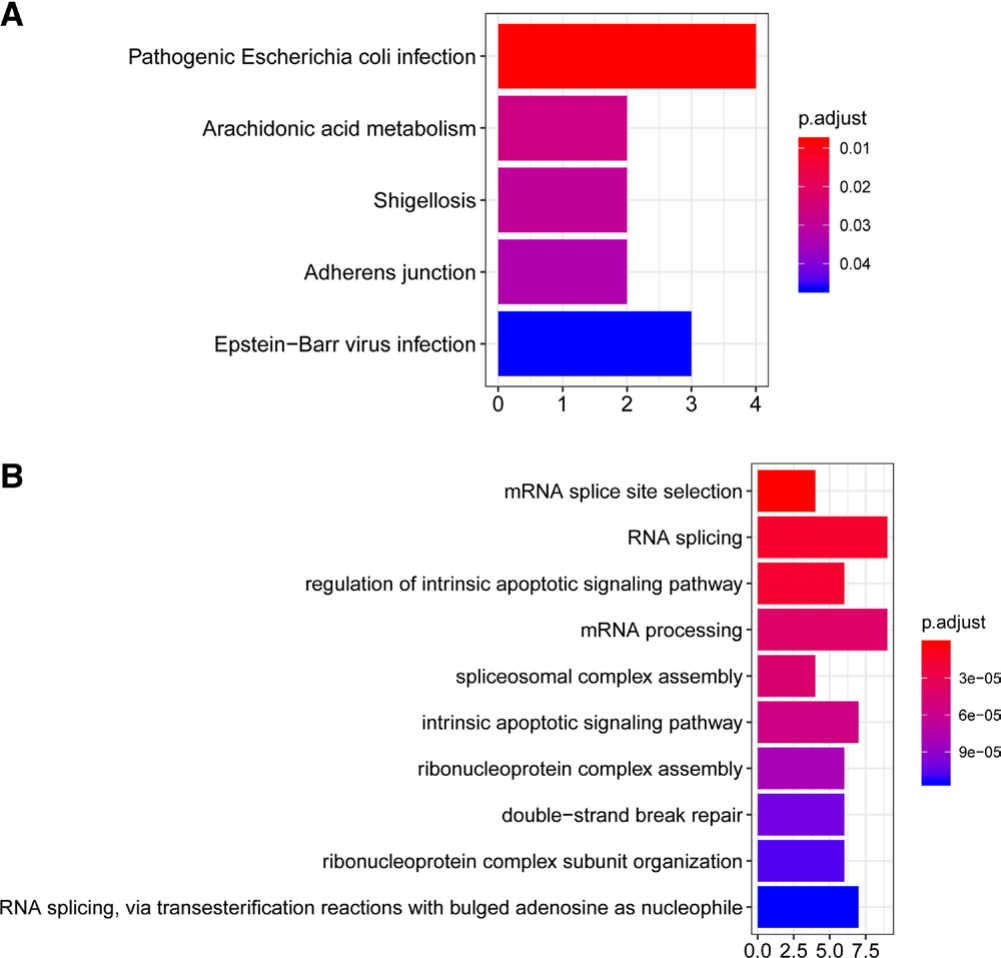
The GO and KEGG pathway enrichment analysis of DEGs. (A) KEGG pathways associated with DEGs. (B) The top 10 biological processes associated with DEGs. DEG = different expressed gene, GO = Gene Ontology, KEGG = Kyoto Encyclopedia of Genes and Genomes.

Functional enrichment analysis showed that upregulated DEGs were enriched in 5 KEGG pathways, including “pathogenic Escherichia coli infection,” “arachidonic acid metabolism,” “adherens junction,” and “EBV infection” (Fig. 2A). GO analysis indicated that upregulated DEGs were enriched in 272 GO biological processes. The top 10 biological processes are shown in Fig. 2B, such as “RNA splicing,” “regulation of intrinsic apoptotic signaling pathway,” “mRNA processing,” and “spliceosomal complex assembly.” However, downregulated DEGs were not enriched in any KEGG pathways and GO biological processes.

### 3.3. PPI network analysis

**Figure 3.**
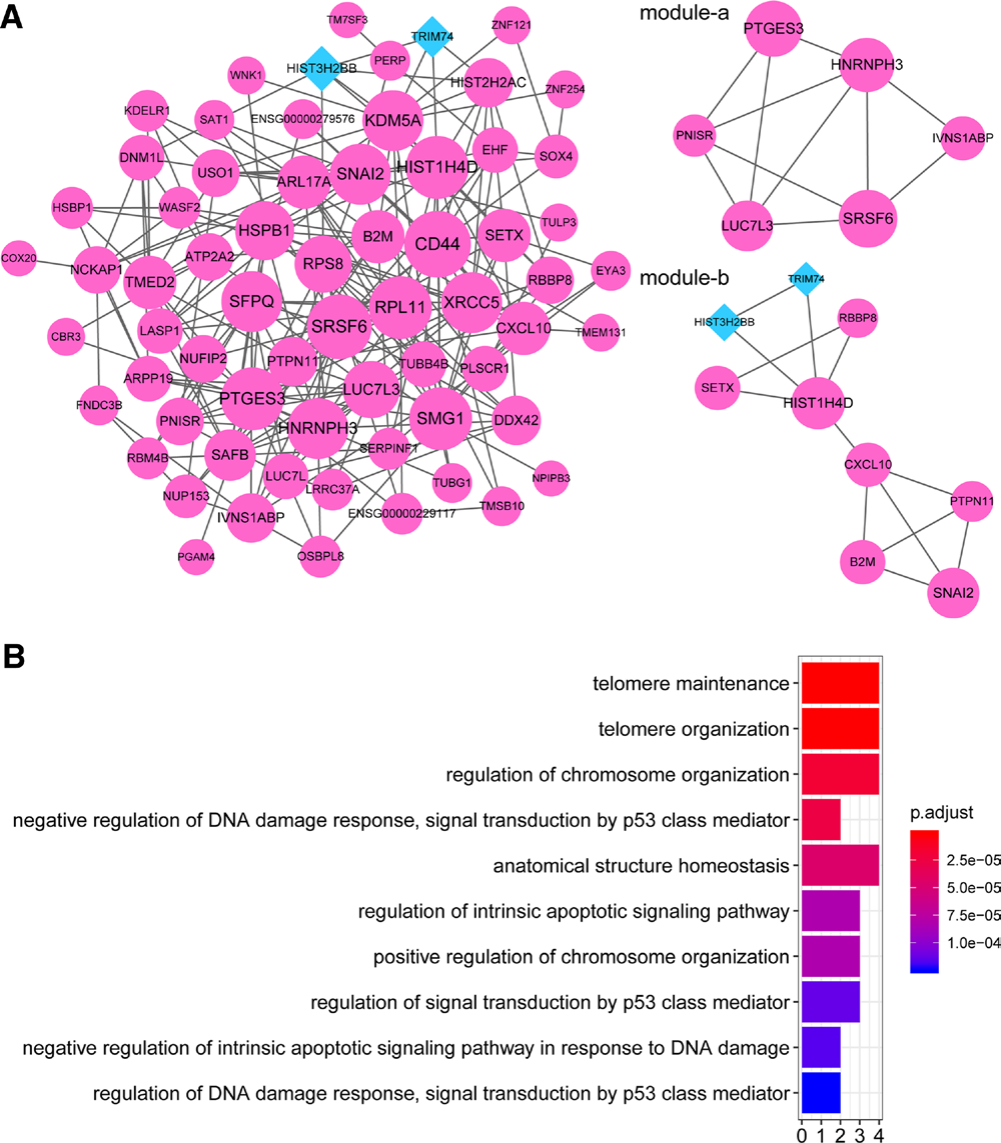
PPI network analysis of DEGs. (A) The PPI network and 2 submodules of DEGs. Red circular node represents upregulated genes and blue prismatic node represents downregulated genes. (B) The top 10 biological processes associated with DEGs in the PPI network. DEG = different expressed gene, PPI = protein–protein interaction.

Overall, 66 nodes and 232 protein interaction pairs were included in the PPI network (Fig. 3A). The degree top 10 nodes are exhibited in Table 1 and were used as hub genes, which mainly contained CD44, SRSF6, RPL11, SMG1, HIST1H4D, PTGES3, SFPQ, XRCC5, SNAI2, and HNRNPH3. These crucial genes were enriched in 102 GO biological processes and the top 10 biological processes in order of *P* value are shown in Figure 3B, inclusive of “telomere maintenance,” “telomere organization,” and “anatomical structure homeostasis.”

**Table 1:** Top 10 genes in the protein–protein interaction network and genes in the submodules (hub gene list).

Degree top 10			Module-a			Module-b		
Nodes	Regulation	Degree	Nodes	Regulation	Degree	Nodes	Regulation	Degree
CD44	UP	17	SRSF6	UP	16	HIST1H4D	UP	15
SRSF6	UP	16	PTGES3	UP	15	SNAI2	UP	14
RPL11	UP	15	HNRNPH3	UP	14	B2M	UP	10
SMG1	UP	15	LUC7L3	UP	12	CXCL10	UP	10
HIST1H4D	UP	15	IVNS1ABP	UP	8	SETX	UP	10
PTGES3	UP	15	PNISR	UP	7	PTPN11	UP	8
SFPQ	UP	14				RBBP8	UP	7
XRCC5	UP	14				HIST3H2BB	DOWN	6
SNAI2	UP	14				TRIM74	DOWN	4
HNRNPH3	UP	14						

**Figure 4.**
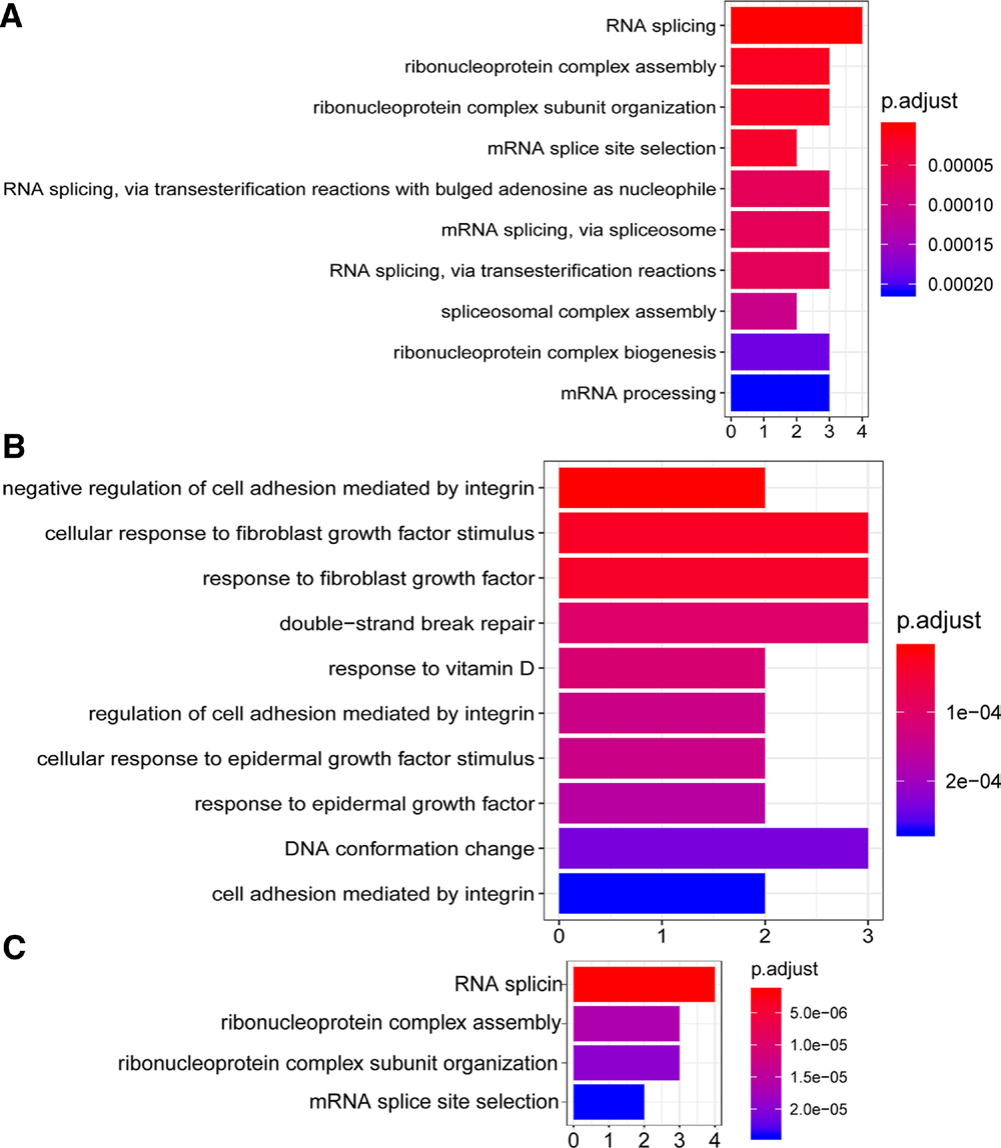
Results of functional enrichment analysis. (A, B) Gene Ontology biological processes associated with nodes in module-a and module-b, respectively. (C) KEGG pathways associated with nodes in module-b. KEGG = Kyoto Encyclopedia of Genes and Genomes.

Furthermore, the PPI network included 2 submodules (Fig. 3A). Specifically, module-a (score = 4.4) contained 6 upregulated genes, which were mainly associated with RNA splicing, ribonucleoprotein complex assembly, and ribonucleoprotein complex subunit organization based on GO analysis (Fig. 4A). Module-b (score = 3.25) included 7 upregulated genes and 2 downregulated genes (Table 1), which were primarily implicated with GO biological processes including “response to fibroblast growth factor”, “response to vitamin D”, and “regulation of cell adhesion mediated by integrin” (Fig. 4B), and 4 KEGG pathways, including “RNA splicing”, “mRNA splice site selection”, and “ribonucleoprotein complex assembly” (Fig. 4C).

### 3.4. The expression patterns of hub genes in NPC tumors with different clinical stages and morphologies

**Figure 5.**
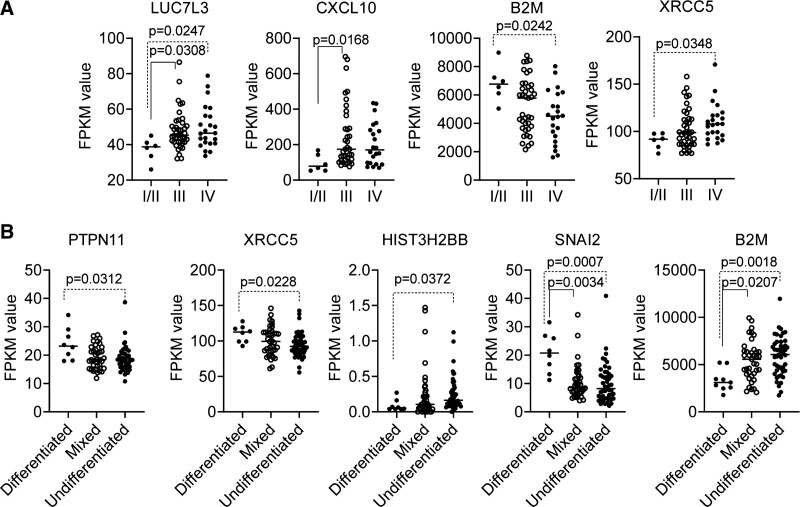
The expression of several hub genes in tumors of different clinical stages and morphologies in the dataset GSE102349. (A), the expression levels (FPKM value) of the genes, *LUC7L3*, *CXCL10*, *B2M*, and *XRCC5* in tumors of different clinical stages. (B) The expression levels (FPKM value) of the genes, *PTPN11*, *XRCC5*, *HIST3H2BB*, *SNAI2*, and *B2M*, in tumors of different morphologies. Difference was identified using the nonparametric Kruskal–Wallis H test with Dunn multiple comparisons test.

Twenty nonoverlapping DEGs, including the top 10 nodes in the PPI network and all nodes in the 2 submodules, were regarded as hub genes (Table 1). The GSE102349 microarray validation showed that only 4 and 5 of the 20 hub DEGs were differentially expressed in tumors in different clinical stages and morphologies, respectively (nonparametric Kruskal–Wallis H test, *P* < .05; Fig. 5A, B). Statistical analysis showed that genes *LUC7L3*, *CXCL10*, and *XRCC5* were upregulated in advanced NPC tumors (III or IV) compared with early-stage tumors (I/II), while the B2M gene was downregulated in advanced NPC tumors (*P* < .05; Fig. 5A). Also, genes *PTPN11*, *XRCC5*, and *SNAI2* were downregulated in mixed (round and spindle) and/or undifferentiated tumors compared with differentiated tumors (*P* < .05; Fig. 5B), while genes *HIST3H2BB* and *B2M* were upregulated in mixed and/or undifferentiated tumors (*P* < .05; Fig. 5B).

### 3.5. Screening of prognostic genes in NPC by univariate Cox regression analysis

The univariate Cox regression analysis was performed to screen factors and hub genes associated with DFS in NPC patients in the GSE102349 dataset. The results showed that stromal tumor-infiltrating lymphocytes (TILs) percent and the expression levels of *CD44*, *B2M*, *PTPN11*, and *TRIM74* were associated with DFS in NPC patients (Table 2). The low level of % stromal TILs (hazard ratio, HR = .980, 95% CI .963–.998, *P* = .029) and *B2M* (HR = 1.000, 95% CI .999–1.000, *P* = .033), and high expression levels of *CD44* (HR = 1.010, 95% CI 1.001–1.020, *P* = .039), *PTPN11* (HR = 1.068, 95% CI 1.012–1.128, *P* = .017), and *TRIM74* (HR = 26.495, 95% CI 1.229–571.371, *P* = .036) were associated with a poor DFS in NPC (Table 2).

**Table 2:** Univariate Cox regression analysis for factors associated with disease-free survival in advanced nasopharyngeal carcinoma in the GSE102349 dataset.

Variables	β	HR	95% CI	*P*
% Stromal TILs	–0.020	0.980	0.963–0.998	.029
% Intratumoral TILs	–0.030	0.970	0.917–1.027	.299
Morphology	–0.653	0.520	0.156–1.731	.287
Nonsynonymous mutation burden	–0.003	0.997	0.992–1.001	.142
Clinical stage (I/II/III/IV)	0.782	2.185	0.894–5.344	.087
CD44	0.010	1.010	1.001–1.020	.039
SRSF6	–0.017	0.984	0.952–1.016	.315
RPL11	0.000	1.000	0.999–1.001	.680
SMG1	–0.016	0.984	0.871–1.112	.798
HIST1H4D	–0.375	0.687	0.044–10.625	.788
PTGES3	0.007	1.007	0.999–1.015	.103
SFPQ	0.025	1.025	0.997–1.054	.080
XRCC5	0.004	1.004	0.979–1.028	.775
SNAI2	–0.019	0.981	0.925–1.040	.981
HNRNPH3	0.014	1.014	0.986–1.043	.321
LUC7L3	–0.006	0.994	0.965–1.025	.703
IVNS1ABP	0.017	1.018	0.992–1.044	.173
PNISR	–0.018	0.982	0.937–1.028	.437
B2M	–0.000291	1.000	0.999–1.000	.033
CXCL10	–0.000478	1.000	0.996–1.003	.766
SETX	–0.063	0.939	0.796–1.108	.457
PTPN11	0.066	1.068	1.012–1.128	.017
RBBP8	–0.023	0.977	0.928–1.029	.386
HIST3H2BB	–0.956	0.384	0.033–4.545	.448
TRIM74	3.277	26.495	1.229–571.371	.036

Morphology is categorized into, differentiated, mixed (round and spindle), and undifferentiated.

CI = confidence interval, HR = hazard ratio, TIL = tumor-infiltrating lymphocytes.

### 3.6. Drug–gene interaction network

**Figure 6.**
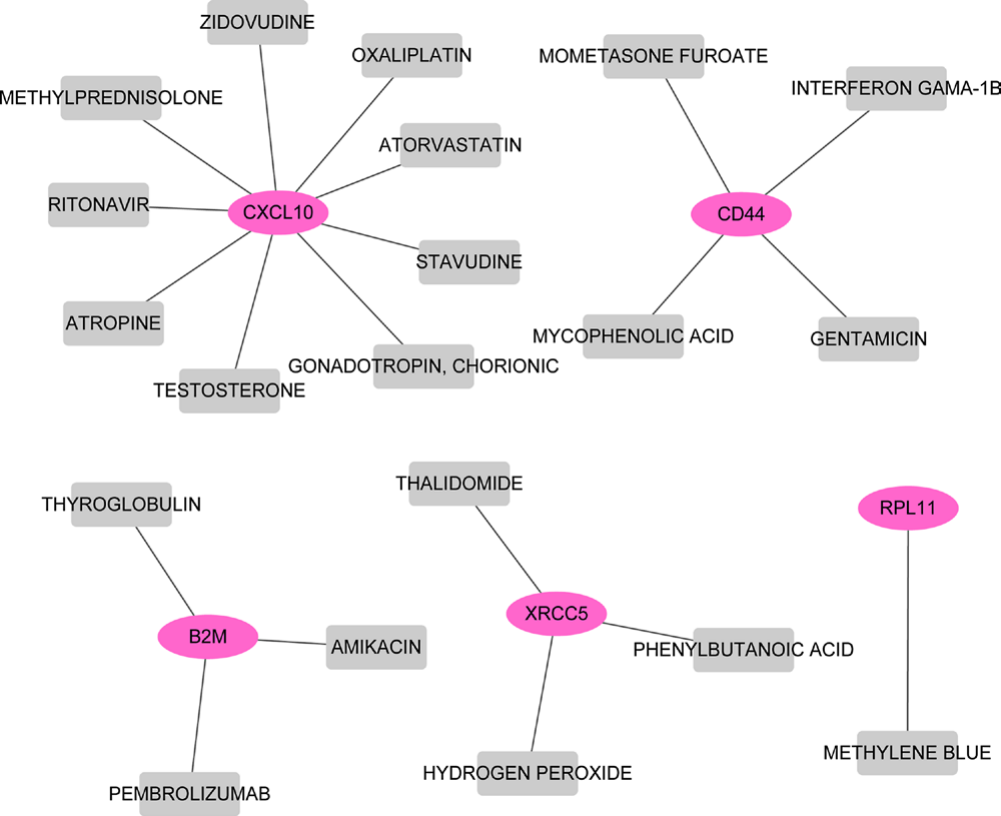
Network of drug–gene interaction. Red circle nodes are upregulated genes; gray square nodes are drug molecules.

Based on the prediction analysis with DGIdb, 20 drug–gene interaction pairs were extracted for the 20 hub genes, which contained 5 upregulated DEGs (*CXCL10*, *CD44*, *B2M*, *XRCC5*, and *RPL11*) and 20 kinds of FDA-approved drug molecules, such as zidovudine, oxaliplatin, methylprednisolone, mometasone furoate, gentamicin, thyroglobulin, pembrolizumab, amikacin, thalidomide, hydrogen peroxide, phenylbutanoic acid, and methylene blue (Fig. 6).

### 3.7. Literature searching of key genes

**Figure 7.**
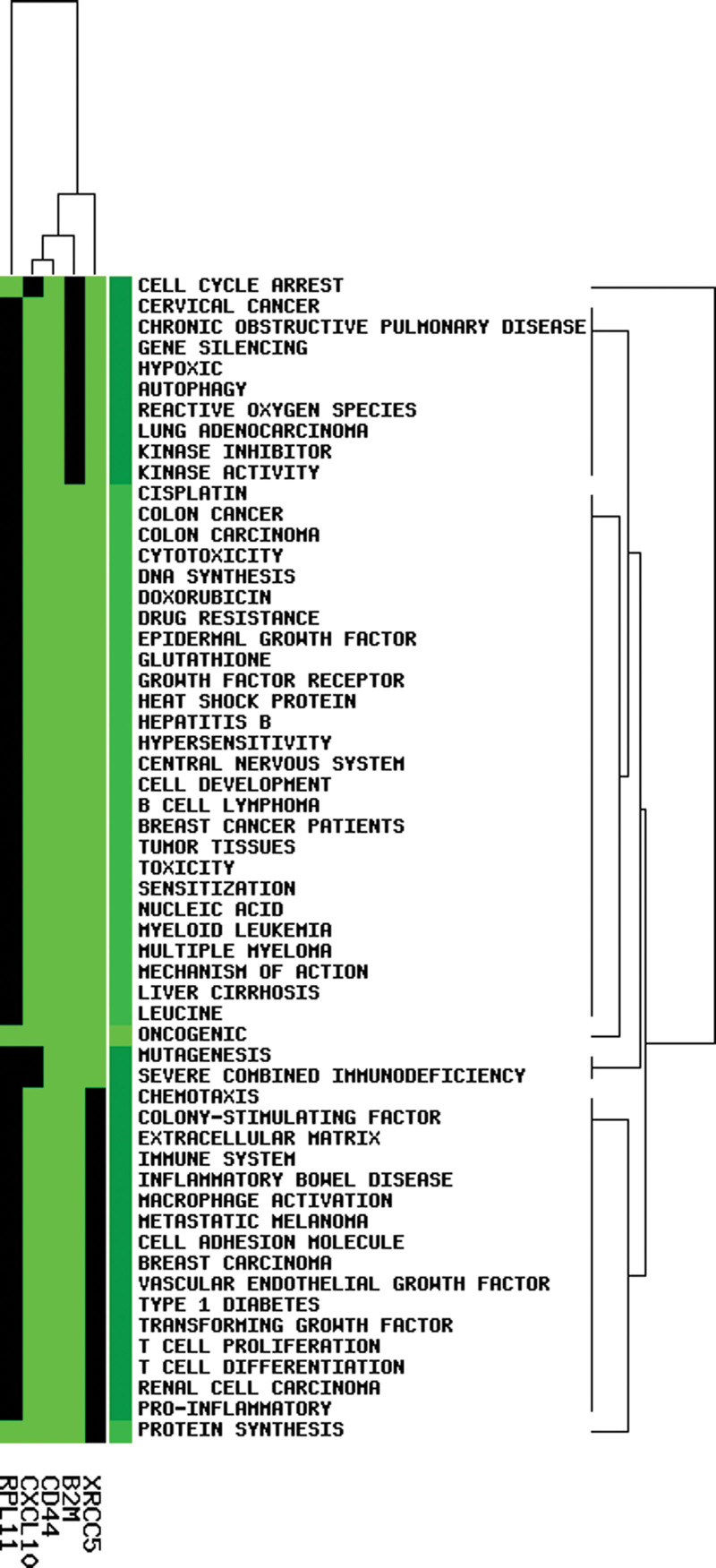
Literature searching of key genes. Heat map of key genes and related functional categories. Green indicates corresponding gene-term association positively reported, black indicates corresponding gene-term association not reported yet.

According to GeneCLiP2.0, the 5 druggable genes, including *RPL11*, *CD44*, *PTPN11*, *XRCC5*, and *B2M*, were associated with multiple biological functions (Fig. 7). Notably, these genes were clustered in the processes or pathways including “cell proliferation,” “cell development,” “hepatitis B virus,” “kinase activity,” “drug resistance,” “autophagy,” and “human cancers” (Fig. 7).

## 4. Discussion

Recently, extensive studies have concentrated on illuminating NPC pathogenesis and the identification of biomarkers for NPC via bioinformatics analyses.^[26,27]^ The present study identified 107 DEGs between LA-NPC tissues with and without distant metastasis after radical treatment. The upregulated DEGs were mainly enriched in cell cycle-related pathways. Hub genes in the PPI network, including *CD44*, *SRSF6*, *RPL11*, *SMG1*, *HIST1H4D*, *PTGES3*, *SFPQ*, *XRCC5*, *SNAI2*, and *HNRNPH3*, were also associated with cell cycle-related pathways, including telomere maintenance and organization. Cox regression analysis showed that the percent of stromal TILs and the expression levels of *CD44*, *B2M*, *PTPN11*, and *TRIM74* were associated with DFS in NPC patients. Genes including *CXCL10*, *B2M*, *XRCC5*, and *PTPN11*, were differentially expressed in NPC tumors of different clinical stages and morphologies. Genes of *CXCL10*, *CD44*, *B2M*, XRCC5, and *RPL11* might be potentially druggable genes for patients with LA-NPC metastasis by regulating various biological functions, including cell cycle, cell proliferation, drug resistance, and autophagy.

Cancer is characterized by the deregulation of cell cycle controls, which leads to the uncontrolled growth of tumor cells.^[28]^ This study found that most of the upregulated DEGs in metastatic NPC tumors were mainly involved in biological processes and pathways related to the cell cycle, such as RNA splicing, mRNA processing, telomere maintenance, and regulation of chromosome organization. As the basic biological process, the cell cycle is defined as the process from the end of a cell division to the end of the next cell division and consists of 2 main events: DNA synthesis and cell division. There are 2 major mechanisms for the regulation of the tumor cell cycle, including cell cycle-driven mechanisms and monitoring mechanisms.^[28]^ The deregulation of cell cycle mechanisms can contribute to the uncontrolled growth of normal cells and conversion to tumor cells.^[28]^ Meanwhile, the uncontrolled cell cycle monitoring mechanisms can also occur in cell proliferation, DNA repair, cell death, etc, thereby causing genomic instability.^[28]^ It is well known that genomic instability is mainly manifested as DNA mutations, deletions, or ectopic, as well as chromosome malformations and aneuploidy, which can result in a burst of mutated genes related to cell cycle regulation.^[28]^ Thus, the key regulators of the cell cycle are promising targets for cancer therapy.^[29]^

There is plenty of research studies that have proved the efficiency of cell cycle-targeted cancer therapy recently.^[30]^ In this study, twenty DEGs including *CXCL10*, *CD44*, *B2M*, *XRCC5*, *PTPN11*, and *RPL11* were identified as hub genes for LA-NPC metastasis. The protein tyrosine phosphatase nonreceptor type 11 (*PTPN11*) gene encodes the SHP-2 phosphatase, which participates in oncogenic events by regulating the expression of genes controlling cell cycle progression, cell proliferation, oncogene-induced senescence, and drug resistance.^[31,32]^ CXC chemokine ligand 10 (CXCL10) is a member of the chemokine family secreted from various cell types.^[33]^ Of note, a previous study has demonstrated that CXCL10 can cause cell cycle redistribution by prolonging G1 and shortening the S phase in cancer cells.^[34]^ Wightman et al^[35]^ showed that coexpression of CXCL10 and CXCR3 predicted an increased risk of metastasis and was associated with poor overall survival and early metastasis. Cluster of differentiation 44 (CD44) is a cell adhesion molecule that is thought as a potential diagnostic tumor marker.^[36]^ The upregulation of CD44 can promote the proliferation and metastasis of tumor cells, while its downregulation inhibits cell cycle progression.^[36]^ A meta-analysis showed that total CD44 isoforms overexpression was correlated with worse overall survival of patients with colorectal cancer, and CD44 expression was related to distant metastasis and poor differentiation.^[37]^ X-ray repair cross-complementing 5 (XRCC5) is known as a DNA repair protein that exerts critical effects on genomic stability and tumorigenesis.^[38]^ Ribosomal protein L11 (RPL11) is essential for cell viability and mutations in the RPL11 gene may contribute to cancer pathogenesis.^[39,40]^ Beta-2-microglobulin (B2M) is a major histocompatibility complex class I molecule that displays antibacterial activity. B2M overexpression is correlated with a poor prognosis in several human cancers, including pancreatic ductal adenocarcinoma^[41–43]^ and it has been considered as a potential target for cancer therapy.^[41,42]^ Consistently, we found that *CXCL10*, *CD44*, *B2M*, *XRCC5*, *PTPN11*, and *RPL11* were upregulated in the LA-NPC tumors with metastasis compared with tumors without metastasis, and genes *CD44*, *B2M*, *PTPN11*, and *RPL11* were associated with the DFS in NPC patients in the GSE102349 dataset.

A study by Mi et al^[26]^ identified 10 molecules, including etomidate, sanguinarine, verteporfin, and chrysin, were associated with NPC metastasis, and are potential drugs for the prevention and treatment of metastasis. However, few clinical trials have been reported for these drugs in NPC. The drug–gene interaction analysis in this study indicated that upregulated genes *CXCL10*, *CD44*, *B2M*, *XRCC5*, and *RPL11* were potential druggable genes that were targeted by >1 FDA-approved drug. For instance, the *CXCL10* gene is targeted by 9 molecules, including zidovudine, oxaliplatin, and methylprednisolone, and the *B2M* gene is targeted by 3 drugs including amikacin, pembrolizumab, and thyroglobulin. Drugs including pembrolizumab, oxaliplatin, methylprednisolone, hydrogen peroxide, and amikacin, have been used in the clinical treatment of NPC.^[44–48]^ These results showed that these genes might be used as promising targets for NPC therapy or metastasis.

The limitation of this study is the lack of validation experiments for expression, metastatic/prognostic value, and druggability of genes, including *CXCL10*, *CD44*, *B2M*, *XRCC5*, *PTPN11*, and *RPL11*. The association of *CD44*, *B2M*, *PTPN11*, and *TRIM74* with the DFS of NPC patients was preliminarily evaluated in the GSE102349 dataset. However, validation using microarray datasets or clinical trials reporting the prognostic values of the hub genes, or experiments confirming their therapeutic value might provide more evidence for the results of this study.

## 5. Conclusions

In conclusion, 107 DEGs between LA-NPC tissue specimens with and without distant metastasis after radical treatment were identified in the dataset of GSE103611. Upregulated expression of *CXCL10*, *CD44*, *B2M*, *XRCC5*, and *RPL11* in LA-NPC tissues after radiotherapy treatment may promote metastasis. The univariate Cox regression analysis showed that the percent of stromal TILs and the expression levels of *CD44*, *B2M*, *PTPN11*, and *TRIM74* were associated with DFS in NPC patients. Five DEGs, including CXCL10, CD44, B2M, XRCC5, and RPL11 might be potential druggable genes for patients with LA-NPC metastasis by regulating cell cycle-related pathways. Monitoring the expression profiles of these genes may provide a reference for clinical practice.

## Author contributions

Conception and design of the research: Rongrong Hu and Lujiao Mo. Acquisition, analysis and interpretation of data: Rongrong Hu, Xujun Xu, Lujiao Mo, Yuxiang Liu, and Mengjie Chen. Statistical analysis: Rongrong Hu and Xujun Xu. Manuscript drafting: Rongrong Hu and Xujun Xu. Manuscript revision for important intellectual content: Lujiao Mo, Mengjie Chen, and Yuxiang Liu. All authors have read and approved the manuscript.
